# Potential Association of Air Leak Syndromes With E-cigarette or Vaping Product Use-Associated Lung Injury (EVALI)

**DOI:** 10.7759/cureus.93395

**Published:** 2025-09-28

**Authors:** Chidi Anakebe, Ragda Abdallah, Mahil Abdalla, Fatima Taha, Haji Sheeraz Khan

**Affiliations:** 1 Department of Pediatrics, Hull University Teaching Hospitals NHS Trust, Hull, GBR; 2 Department of Emergency Medicine, Hull University Teaching Hospitals NHS Trust, Hull, GBR

**Keywords:** adolescent vaping, air leak syndrome, e-cigarette or vaping-associated lung injury, spontaneous pneumomediastinum, subcutaneous emphysema

## Abstract

E-cigarette or vaping product use-associated lung injury (EVALI) has emerged as a public health concern, with most reports describing acute respiratory illness characterized by bilateral infiltrates and hypoxemia. Air leak syndromes, such as spontaneous pneumomediastinum and subcutaneous emphysema, are not yet widely recognized as part of the EVALI spectrum. We present the case of a 15-year-old male with a history of mild asthma who developed extensive subcutaneous emphysema, pneumomediastinum, and pneumorrhachis following the use of vaping products and cannabis inhalation. His presentation included acute respiratory distress, hypoxemia, and swelling of the neck and face. Imaging confirmed widespread air leak without evidence of esophageal perforation. He was managed conservatively with oxygen therapy, total parenteral nutrition, antibiotics, and supportive care. The patient made a full recovery and was discharged with outpatient follow-up. A targeted literature search identified several reports linking vaping to spontaneous air leak syndromes, though most involved young adults. To our knowledge, this represents one of the youngest reported cases, highlighting a potentially underrecognized complication of vaping in adolescents. This case underscores the importance of considering vaping-associated pneumomediastinum in young patients presenting with chest pain and respiratory distress. With vaping prevalence rising sharply among adolescents, clinicians should actively inquire about e-cigarette and cannabis use in such presentations. Further research is needed to clarify the mechanisms, risk factors, and long-term outcomes of vaping-related air leak syndromes.

## Introduction

Smoking (including vaping) accounts for about 80,000 preventable deaths annually in the UK and is responsible for one in four cancer deaths each year, costing the UK government approximately £17 billion per year [1]. The use of e-cigarettes and vaping has risen sharply in the past three years, with 20.5% of children aged 11-17 having tried vaping, compared with 15.8% in 2022 [1,2]. There has been a 50% increase in children in the “experimentation” group (tried once or twice), from 7.7% in 2022 to 11.6% in 2023, a trend observed over recent years [2].

The rise in vaping and e-cigarette use in the past decade has led to recognition of a new disease entity, e-cigarette or vaping product use-associated lung injury (EVALI). This was declared an outbreak in the USA in March 2019 and was first reported in Europe a year later, with significant morbidity and mortality [3,4]. Research and data on e-cigarette use remain limited, and the long-term sequelae and impacts on children are unclear [5].

Here, we describe a rare acute complication of vaping in a 15-year-old male who presented with spontaneous air leak syndrome following the use of vaping products. He was managed conservatively and achieved a good clinical outcome.

## Case presentation

A 15-year-old adolescent male presented to the ED with respiratory distress and low oxygen saturation. He was tachypneic (respiratory rate 24/min), lethargic, and hypoxic (oxygen saturation 86% on room air), requiring face mask oxygen to maintain saturations during ambulance transfer. He had a dry cough for one week and a fever for four days. On the morning of the presentation, he experienced several episodes of vomiting, after which he developed sharp, stabbing central chest and back pain. His mother also reported swelling of the left side of his neck and face that began that same morning.

He had a previous history of bronchiolitis in infancy, which had resolved without residual lung disease on follow-up. He also had mild asthma, controlled with occasional use of a salbutamol inhaler. He had not undergone any intervening chest imaging before this presentation. There was no family history of spontaneous pneumothorax or connective tissue disorders. He admitted to vaping and inhaling cannabis, reporting that he had been vaping for the past year and consumed approximately 3,500 puffs per week.

On arrival to the ED, his heart rate was 100 beats per minute, blood pressure 122/76 mmHg, capillary refill time <2 seconds, and temperature 37 °C. He appeared mottled, and examination revealed palpable crepitus on the left side of the neck, chest, and abdomen. He had good bilateral air entry with normal breath sounds. He received 15 liters of oxygen via a non-rebreathing mask to maintain saturations at 100%, a fluid bolus of 10 ml/kg, and a stat dose of cefotaxime.

**Figure 1:**
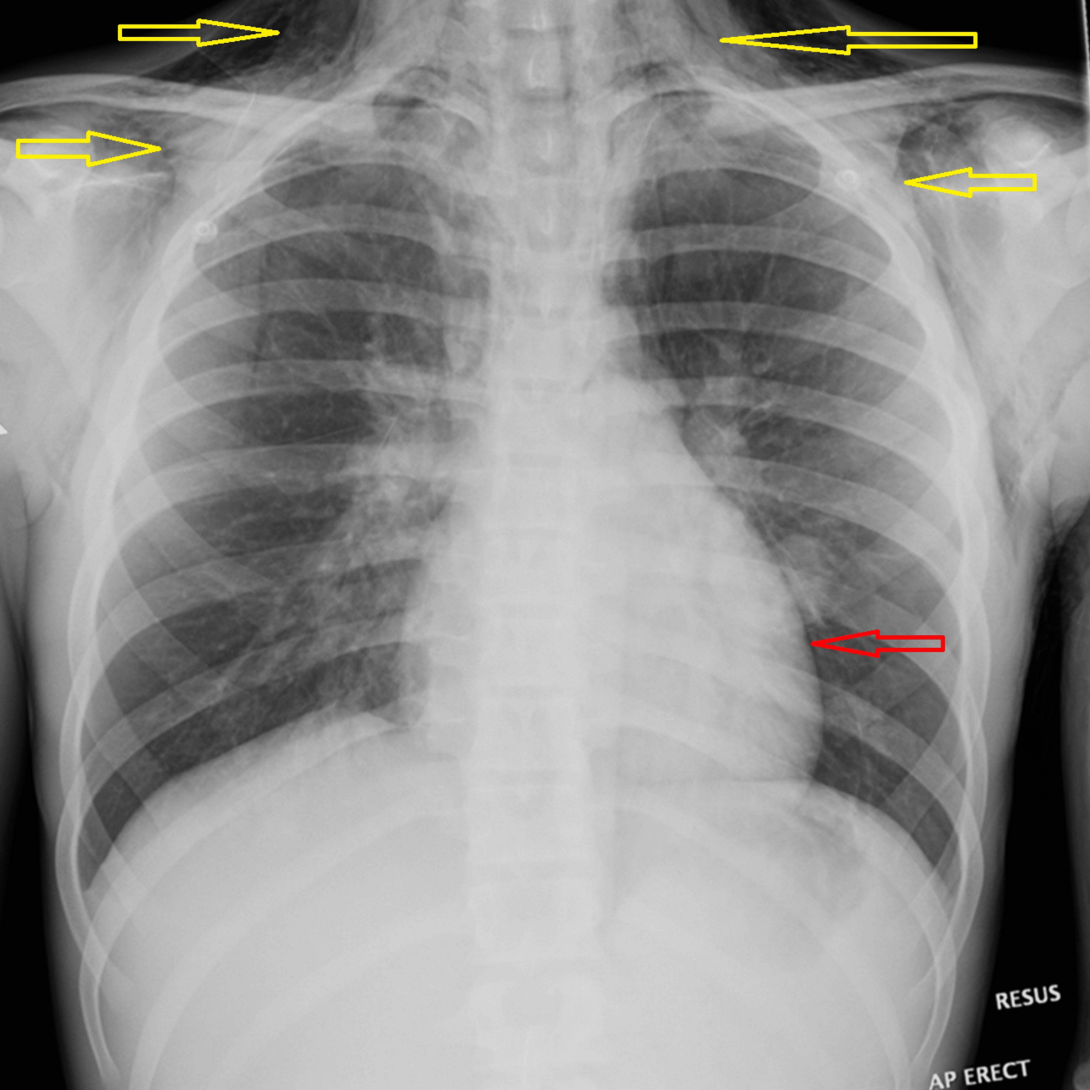
Chest X-ray AP erect chest radiograph showing diffuse subcutaneous emphysema (yellow arrows) tracking along the soft tissues of the neck and upper chest wall, with associated pneumomediastinum (red arrow).

His chest X-ray on admission demonstrated diffuse subcutaneous emphysema, most prominent around the neck and upper chest wall, with associated pneumomediastinum (Figure 1).

**Figure 2:**
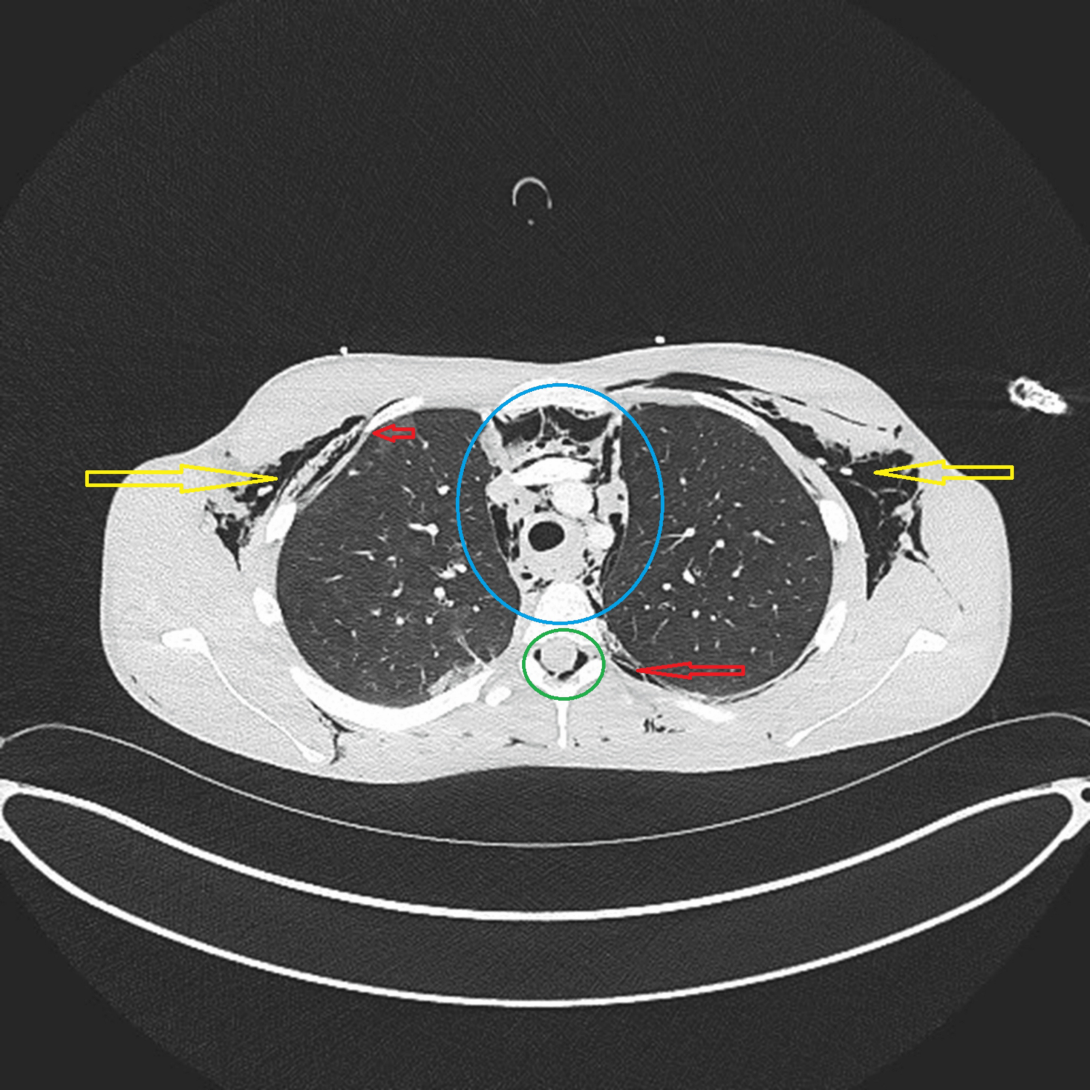
Contrast-enhanced CT chest Axial CT image demonstrating extensive subcutaneous emphysema (yellow arrows) and pneumomediastinum (blue circle). Pneumorrhachis is visible (green circle), and pneumothorax is highlighted (red arrows).

Furthermore, a contrast-enhanced CT scan of the chest revealed extensive subcutaneous emphysema, pneumomediastinum, pneumothorax, and pneumorrhachis, with no evidence of esophageal perforation or extraluminal contrast leakage (Figure 2). The CT report also noted consolidation in the left lower lobe and subpleural ground-glass opacities in the right upper lobe, although some findings were not clearly visible on the provided representative images.

Subsequently, he was admitted to the Pediatric High Dependency Unit for monitoring. He did not tolerate high-flow nasal cannula oxygen, so he was continued on 60% oxygen via face mask to maintain oxygen saturations above 95%. He was managed with total parenteral nutrition, empirical antibiotics (IV co-amoxiclav and oral clarithromycin, to which the organisms were later confirmed sensitive), omeprazole, and analgesia, and was kept nil by mouth for a total of seven days due to potential concerns of esophageal perforation.

His blood results showed hemoglobin 162 g/L, white cell count 7.1 × 10⁹/L, platelets 149 × 10⁹/L, and CRP 148 mg/L. Urine toxicology was positive for cannabinoids. It was not documented whether he continued vaping or inhaling cannabis during the symptomatic period before admission; given that cannabinoid metabolites can remain detectable in urine for several days to weeks in regular users, the positive result could not distinguish between ongoing and prior use. Respiratory PCR was positive for adenovirus, and sputum culture grew *Haemophilus influenzae *and Group A streptococcus.

A repeat barium swallow after 10 days did not show any evidence of esophageal leak. He was restarted on oral feeds, and total parenteral nutrition was discontinued on day 8 of admission. He required 2.5 days of oxygen therapy during his admission and received a total of 1 week of antibiotics before being discharged with a three-week course of oral omeprazole and outpatient pediatric follow-up.

The patient’s vital signs and laboratory investigations are summarized in Table 1.

**Table 1:** Patient’s vital signs and laboratory investigations ALP, alkaline phosphatase; ALT, alanine aminotransferase; APTT, activated partial thromboplastin time; bpm, beats per minute; Hb, hemoglobin; POCT, point-of-care testing; PT, prothrombin time; RSV, respiratory syncytial virus; WCC, white cell count

Parameter	Result	Reference range
Vital signs		
Heart rate	100 bpm	60-100 bpm
Blood pressure	122/76 mmHg	90-120/60-80 mmHg
Respiratory rate	24 breaths/min	12-20 breaths/min
Oxygen saturation (room air)	86%	>95%
Temperature	37 °C	36-37.5 °C
Arterial blood gas		
pH	7.41	7.35-7.45
pCO₂	6.3 kPa	4.5-6.1 kPa
pO₂	8.2 kPa	12-15 kPa
Standard bicarbonate (HCO₃⁻)	24 mmol/L	22-26 mmol/L
Base excess	+4 mmol/L	-2 to +2 mmol/L
Oxygen saturation (blood gas)	27%	>95%
Sodium (Na⁺)	132 mmol/L	135-144 mmol/L
Potassium (K⁺)	4.5 mmol/L	3.5-5.3 mmol/L
Ionized calcium (Ca²⁺)	1.14 mmol/L	1.15-1.27 mmol/L
Glucose (POCT)	4.0 mmol/L	3.6-5.2 mmol/L
Lactate	1.5 mmol/L	0-1.3 mmol/L
Hemoglobin (blood gas)	162 g/L	135-175 g/L
Chloride	93 mmol/L	96-106 mmol/L
Full blood count		
Hemoglobin	162 g/L	135-175 g/L
WCC	7.1 × 10⁹/L	4.0-13.0 × 10⁹/L
Platelets	149 × 10⁹/L	150-400 × 10⁹/L
Neutrophils	5.77 × 10⁹/L	2.0-7.0 × 10⁹/L
Lymphocytes	0.82 × 10⁹/L	1.2-5.2 × 10⁹/L
Monocytes	0.49 × 10⁹/L	0.2-0.8 × 10⁹/L
Eosinophils	0.10 × 10⁹/L	0.04-0.40 × 10⁹/L
Basophils	0.02 × 10⁹/L	0.01-0.10 × 10⁹/L
Urea and electrolytes		
Sodium	131 mmol/L	135-144 mmol/L
Potassium	4.2 mmol/L	3.5-5.3 mmol/L
Chloride	102 mmol/L	96-109 mmol/L
Bicarbonate	27 mmol/L	24-32 mmol/L
Urea	6.0 mmol/L	3.0-7.6 mmol/L
Creatinine	63 µmol/L	45-75 µmol/L
Liver function tests		
Bilirubin	9 µmol/L	<21 µmol/L
ALP	85 IU/L	75-400 IU/L
ALT	62 IU/L	5-45 IU/L
Albumin	39 g/L	36-48 g/L
Total protein	80 g/L	60-80 g/L
Inflammatory markers and coagulation		
CRP	148 mg/L	0-8 mg/L
PT	10.3 seconds	9.5-12.0 seconds
APTT	24.6 seconds	26-29 seconds
Microbiology		
Respiratory PCR	Adenovirus positive; SARS-CoV-2, influenza A/B, RSV, and other pathogens negative	-
Sputum culture	*Haemophilus influenzae*, Group A streptococcus	-
Blood culture	No growth at 36 hours	-
Toxicology		
Urine toxicology	Cannabinoids detected	-

## Discussion

Overview of EVALI and air leak syndromes

EVALI is a diagnosis of exclusion, presenting with varied respiratory features [6]. Vitamin E acetate has been implicated in some cases of EVALI [7], and bilateral ground-glass opacities are among the most frequent CT findings [8]. In our patient, CT demonstrated unilateral changes rather than the typical bilateral distribution, limiting direct comparison with previously described cases of EVALI. Furthermore, the concurrent detection of adenovirus, *H. influenzae*, and Group A streptococcus means that infection cannot be fully excluded as an alternative or contributory explanation for the air leak syndrome. Although less commonly recognized, spontaneous air-leak phenomena such as pneumomediastinum, pneumorrhachis, and subcutaneous emphysema may occur, as illustrated by this adolescent case.

Proposed mechanisms of injury

The Macklin effect, alveolar rupture with air tracking along bronchovascular sheaths, likely explains these findings. Barotrauma from deep inhalation against a closed glottis (Valsalva maneuver) or after forced exhalation (Müller maneuver) can precipitate this [9,10]. Cannabis use may potentiate such pressure swings [11].

It is important to note that this patient also had infectious cofactors. Respiratory PCR was positive for adenovirus, and sputum culture grew *H. influenzae* and Group A streptococcus. Each of these pathogens has been reported in association with spontaneous subcutaneous emphysema and pneumomediastinum. A case report by Alnofal et al. described a young adult who developed extensive subcutaneous emphysema and pneumomediastinum in the context of influenza B infection [12]. Similarly, Duvekot et al. reported a complicated course of mediastinal emphysema following Group A beta-hemolytic streptococcal infection after adenotonsillectomy [13]. Viral and bacterial respiratory infections may therefore have contributed to alveolar fragility in this patient, acting as cofactors alongside barotrauma from vaping and cannabis inhalation.

Comparison with reported cases

SPM occurs in 1-14 per 1,000 hospitalized patients, mostly in young men [14]. Prior reports of vaping-associated SPM predominantly involved young adults [11,13,14]. Weiss et al. described marijuana users with SPM (mean age 22.5 years) [11], and Adhikari et al. reported a 24-year-old with vaping-induced SPM managed conservatively [15]. Li and Miller described a 16-year-old footballer with vaping-related emphysema [16].

Implications for clinical practice

Adolescents presenting with chest pain or unexplained respiratory distress should be specifically asked about vaping and cannabis use. Imaging is essential for diagnosis. Most cases are benign and self-limiting, with conservative management sufficient, although multidisciplinary care may be required in severe presentations [14,15].

Limitations and future directions

Causality cannot be proven in a single case. Unlike many reports of EVALI, this patient did not demonstrate bilateral ground-glass opacities, which limits direct comparison with previously described patterns. In addition, the concurrent detection of adenovirus, *H. influenzae*, and Group A streptococcus means that infection cannot be excluded as a possible cause or contributing factor for the air leak syndrome. The precise agents in vaping products also remain unclear, although vitamin E acetate has been implicated in some cases [7]. Larger studies are needed to define mechanisms, assess how infectious cofactors interact with vaping, and guide adolescent-specific management [6-8,11-13,15,16].

## Conclusions

This case suggests that vaping may be associated with spontaneous air leak syndromes in adolescents, although causality cannot be established from a single report. The findings of extensive subcutaneous emphysema, pneumomediastinum, and pneumorrhachis in such a young patient highlight a potentially underrecognized complication of vaping and cannabis inhalation.

Clinicians should consider vaping in the differential diagnosis of adolescents presenting with chest pain and respiratory distress, particularly when air-leak phenomena are identified on imaging. As vaping use continues to rise among young people, further studies are needed to explore potential mechanisms, identify risk factors, and guide appropriate management strategies in this population.
